# Self-Adaptive Spike-Time-Dependent Plasticity of Metal-Oxide Memristors

**DOI:** 10.1038/srep21331

**Published:** 2016-02-19

**Authors:** M. Prezioso, F. Merrikh Bayat, B. Hoskins, K. Likharev, D. Strukov

**Affiliations:** 1 Department of Electrical and Computer Engineering, University of California at Santa Barbara, Santa Barbara, CA 93106, United States; 2 Department of Physics and Astronomy, Stony Brook University, Stony Brook, NY 11794, United States

## Abstract

Metal-oxide memristors have emerged as promising candidates for hardware implementation of artificial synapses – the key components of high-performance, analog neuromorphic networks - due to their excellent scaling prospects. Since some advanced cognitive tasks require spiking neuromorphic networks, which explicitly model individual neural pulses (“spikes”) in biological neural systems, it is crucial for memristive synapses to support the spike-time-dependent plasticity (STDP). A major challenge for the STDP implementation is that, in contrast to some simplistic models of the plasticity, the elementary change of a synaptic weight in an artificial hardware synapse depends not only on the pre-synaptic and post-synaptic signals, but also on the initial weight (memristor’s conductance) value. Here we experimentally demonstrate, for the first time, an STDP behavior that ensures self-adaptation of the average memristor conductance, making the plasticity stable, i.e. insensitive to the initial state of the devices. The experiments have been carried out with 200-nm Al_2_O_3_/TiO_2−*x*_ memristors integrated into 12 × 12 crossbars. The experimentally observed self-adaptive STDP behavior has been complemented with numerical modeling of weight dynamics in a simple system with a leaky-integrate-and-fire neuron with a random spike-train input, using a compact model of memristor plasticity, fitted for quantitatively correct description of our memristors.

In biological neural systems, neurons communicate with each other with action potential pulses - “neural spikes^1^”. While some of the network activity information is encoded in the average spiking rate, many experiments in neurobiology suggest that the timing of individual spikes matters, and is essential for coordinated processing of temporal and spatial information^2^,^3^. Indeed, encoding information with single spikes or inter-spike intervals provides a higher information capacity than the firing-rate codes which represent only the average spiking activity^4^. These observations motivated the development of spiking neuromorphic hardware circuits which explicitly model neural spikes^1^,^4^. An additional motivation^5^,^6^,^7^ for pursuing spiking neuromorphic networks is their higher energy efficiency, recently demonstrated in a very large system^8^.

In the simplest spiking neuromorphic networks, each neuron is modeled as a leaky-integrate-and-fire unit, which integrates incoming spikes and fires its own spike when the integrated action potential reaches a certain threshold^1^. The fired spike, weighed according to the strengths of the corresponding synapses, is applied to the input of other neurons. Additionally, the fired spike is also propagated backwards to the input synapses to provide their weights’ adaptation – for example, according to the spike-timing-dependent-plasticity (STDP) rule^9^,^10^,^11^,^12^, which ensures Hebb-like learning^9^. For the most common STDP type, found for example in Layer 5 of the neocortex^13^, the synaptic weight is increased if the post-synaptic spike follows soon after the pre-synaptic one (implying their causal relation), is decreased if their timing order is opposite (implying a random coincidence of the spikes), and is virtually unaltered if the time interval Δ*t* between the spikes is larger than a few milliseconds.

The STDP-enabling hardware based on traditional integrated circuit technologies, in which synaptic weight values are stored, for example, in digital static-random access memories^8^, or as analog charges in switched capacitor structures^7^, can hardly ensure the network density necessary for cortex-scale systems. On the other hand, the values may be stored as conductivities of very compact, two-terminal, nonvolatile devices, “memristors”, which may be scaled down to ensure such density^14^,^15^,^16^. This is why, following several suggestions of STDP implementation in memristors, using various shapes of pre- and post-synaptic pulses^17^,^18^,^19^,^20^,^21^,^22^, there has been a recent surge of experimental demonstrations of the STDP functionality in organic^23^,^24^,^25^,^26^, complex-oxide^27^, sulfide^28^,^29^, silicon-oxide^30^, hafnium-oxide^31^ and phase-change^32^ devices.

In this work we have shown that in metal-oxide memristors STDP may be self-adaptive, excluding the need in the continuous adjustment of the average conductance of each device.

## Results

**Figure 1:**
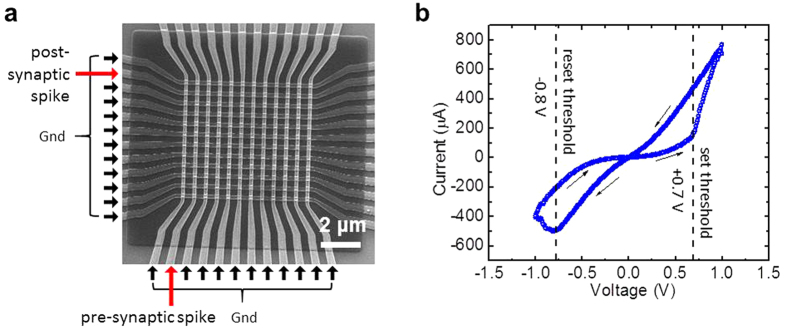
Metal-oxide memristive devices. (**a**) SEM image of the active area of a memristive crossbar, with the particular inputs used in the STDP experiment shown on the margins. (**b**) Typical *I-V* curve of a memristor after its forming, with the dashed lines indicating the effective set and reset thresholds.

All experiments were carried out with Pt/Al_2_O_3_/TiO_2−x_/Ti/Pt memristors integrated in 12 × 12 crossbar circuits (Fig. 1a) – see the Methods section for fabrication details. Figure 1b shows a typical switching hysteresis of a crossbar-integrated memristor at a quasi-DC symmetric voltage sweep. The ON/OFF current ratio measured at a non-disturbing bias of 0.2 V is close to 10. The results of a detailed electrical characterization of these crossbar-integrated devices, including their switching endurance of at least 5,000 cycles, projected retention time in excess of 10 years, and low variability of forming and switching voltages, were reported earlier^33^.

**Figure 2:**
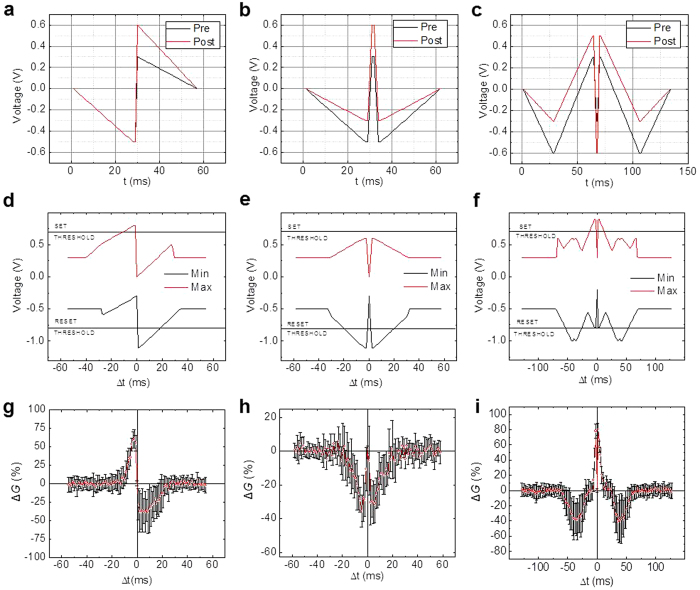
Experimental results for spike-time-dependent plasticity. Implemented STDP windows similar to those typical for biological synapses: in Layer 5 (the left column) and Layer 4 (the middle column) of the neocortex, and in GABAergic synapses (the right column). (**a**–**c**) The used shapes of pre-synaptic (“pre”, black lines) and post-synaptic (“post”, red lines) voltage pulses. (**d**–**f**) The time maxima and minima of the net voltage applied to the memristor, as functions of the time interval Δ*t* between the pre- and post-synaptic pulses. (**g**–**i**) The experimentally measured STDP windows, i.e. the changes of memristor’s conductance as functions of Δ*t*. The red points and black error bars show, respectively, the averages and the standard deviations of the results over 10 experiments for each value of Δ*t*. In these experiments, the initial memristor conductance *G*_0_ was always close to 33 μS.

In the first set of experiments, we have implemented three different biologically-plausible “STDP windows”, i.e. the dependences of the synaptic weight change on the time interval Δ*t* between the pre- and post-synaptic spikes (Fig. 2). In particular, getting each experimental point shown on the bottom panels (g, h, i) of Fig. 2 involved three steps. First, memristor’s conductance *G*, which represents its synaptic weight and was measured at 0.2 V, was set to an initial value *G*_0_ ≈ 33 μS, using a simple but efficient tuning algorithm^34^. Pre-synaptic and post-synaptic pulses of the waveforms shown on one of the top panels (a, b, c) of Fig. 2, selected following the recommendations of Ref. 21, were then applied to the top and bottom wires leading to the selected memristor inside the crossbar, with a certain delay Δ*t* between the pulses, while the remaining lines of the crossbar were kept grounded. Finally, after the pulse application, the new value of memristor’s conductance was measured and its change calculated. The experiment was repeated 10 times for each particular Δ*t*, every time resetting the device to the same initial conductance with 10% accuracy. As Fig. 2 shows, these three different spike shapes result in three different representative STDP window shapes found in various biological synapses^13^. Other window shapes, e.g., those which correspond to Δ*t* sign flip (and hence may be used for the anti-Hebbian rule implementation), may be readily obtained by changing switching polarity and/or modifying pulse timings.

**Figure 3:**
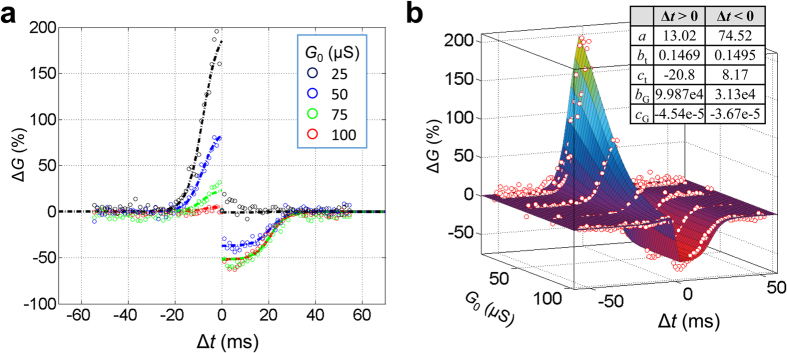
Modeling spike-time-dependent plasticity. (**a**) The experimentally measured STDP window function (circles), for the waveforms shown in Fig. 2a, for several initial values *G*_0_ = 25, 50, 75 and 100 μS together with the results of its fitting with Eqs. (1 (dash-dot lines) and (**b**) the resulting 3D surface. The inset table in panel (**b**) shows the used fitting parameters.

The initial conductance of 33 μS, chosen for the described set of experiments, crudely corresponds to the geometrical mean of two extreme conductance values for the considered memristors. In the second set of tests, the experiment with waveforms corresponding to the first STDP window (Fig. 2g) was repeated for several different initial values *G*_0_ of conductance, spanning the whole dynamic range of our memristors. As this Fig. 3a shows, that STDP for *G*_0_ = 33 μS is balanced, i.e. the maximum change in conductance is roughly the same for positive and negative Δ*t* values. On the other hand, there is no increase in conductance when its initial value is close to its maximum value, and no decrease in conductance in the opposite case, i.e. when *G*_0_ is close to device’s minimum conductance. Such saturation in the switching dynamics is typical for many types of memristors^15^,^16^,^33^,^34^,^35^.

This strong dependence of memristor’s plasticity on its initial state might cause concerns about the possible need in continuous external tuning of each device, which would make large-scale spiking networks impracticable. To investigate this issue, we have carried out numerical simulation of the STDP adaptation, using an analytical, phenomenological (“compact”) model of the experimentally observed conductance change for the particular STDP window shown in Fig. 2g. It has turned out that the change is well described by the following product:


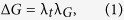



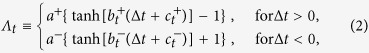



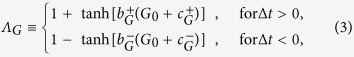


where *a*, *b*, *c* and *d* are fitting parameters. (In some publications^36^,^37^, such functions are called “multiplicative”; note, however, that though at each of the two intervals of Δ*t*, Δ*G* is indeed a product of separate functions of *G*_0_ and Δ*t*, globally it is not, since according to Eq. (3), function Λ_*G*_ depends not only on *G*_0_, but also on Δ*t* – via its sign. Due to this reason, the plots of Δ*G* as a function of Δ*t*, shown with the continuous surface in Fig. 3b, are not globally self-similar.) As the dots in Fig. 3 show, this function, with an appropriate choice of the fitting parameters, describes the experimentally observed behavior very reasonably - see also Fig. S1 and its discussion in Supplementary Information for additional details. Moreover, we believe that such *G*_0_-dependent STDP behavior may be expected for many types of memristive devices with saturating switching dynamics^15^,^16^,^33^,^34^,^35^.

**Figure 4:**
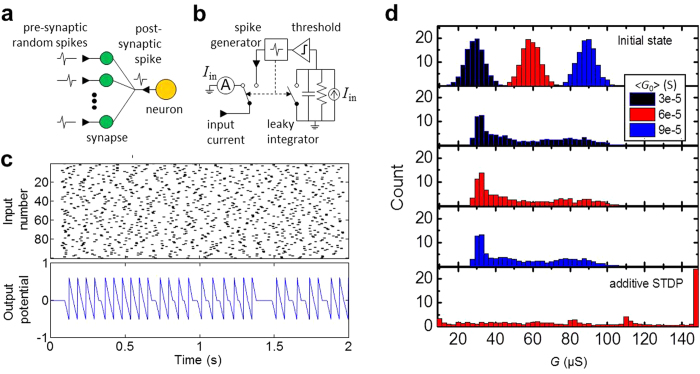
Self-adaptation of spike-time-dependent plasticity. Simulation of memristor self-tuning in a simple spiking network, using Eqs. (1 for STDP description. (**a**) The simulated network; (**b**) its equivalent circuit; (**c**) typical input and output spiking activity; and (**d**) the initial and final distributions of conductances, averaged over 10 runs, for several values of the initial conductance *G*_0_. On panel (**c**), the top graph uses grey color coding to shows the spike initiation times. On panel (**d**), three middle figures show the final distribution of conductances for three values of *G*_0_, after 60 s of simulated time. The bottom figure of panel (**d**) shows the final weight distribution for the hypothetical “additive” STDP model, obtained by artificially setting λ_*G*_ = 1. The neuron parameters are as follows: *R* = 4 kΩ, *C* = 1 μF, activation threshold *U*_*t*_ = 0.5 V.

Using the STDP model so verified, we have simulated the time evolution of memristor conductances in a simple, generic neuromorphic network with just one soma, described with the leaky-integrate-and-fire model, and 100 input synapses (Fig. 4). The network was fed with similar spikes of the shape shown with black lines in Fig. 2a, with random, independent, Poisson-distributed initiation times with 14 Hz average spiking rate. As Fig. 4d shows, memristor conductances eventually evolve to a stable bell-curve distribution independent of their initial values, with the peak of the distribution centered in the intermediate value of the dynamic range. This is not quite surprising, because our model qualitatively corresponds to the typical STDP behavior observed in biology^11^,^38^, and also to phenomenological “multiplicative” models that predict similar self-adaptation^36^,^37^, which is deemed necessary for long-term stability of spiking neural networks. (As illustrated by the bottom panel of Fig. 4d, so-called “additive” STDP models, in which Δ*G* is independent of *G*_0_^36^, cannot ensure such self-adaptation.)

## Discussion

The demonstrated dependence of the STDP window on the applied voltage waveforms (cf. panels (a–c) and (g–i) of Fig. 2) may be readily explained by taking into account that memristor’s conductance changes mostly when the net voltage applied to the device exceeds certain switching threshold voltage – see the dashed lines in Fig. 1b and panels (d–f) of Fig. 2. As the result, the conductance change Δ*G* semi-quantitatively follows either the time maximum or the time minimum of the applied waveforms – whichever exceeds the corresponding threshold more. Panels (d–f) show these voltage extremal values for the used waveforms (a–c); their comparison with the corresponding experimental STDP windows shown in panels (g–i) indeed confirms their similarity. Some slight deviations from this correspondence, for example, the time asymmetry of the window shown on panel (i), may be readily explained by the switching dynamics dependence on the conductive state of the memristor.

Another unexpected anomaly of the data is the presence of the (weak and broad) second peak in the distribution of final conductances in the numerical simulation of synaptic self-adaptation – see Fig. 4d. This peak might be suppressed by balancing device’s asymmetry by slightly varying parameters of the STDP – see Fig. S2 and its discussion.

## Summary

In conclusion, we have experimentally demonstrated that Al_2_O_3_/TiO_2_-based memristors may be used to implement the spike-time dependent plasticity with STDP window shapes similar to those observed in biological neural systems. By fitting the experimental data with a simple compact model, we have shown that such STDP behavior enables self-adaptation of the synaptic weights to a narrow interval in the intermediate value of their dynamic range, at least in a simple (but very representative) spiking network. These results give every hope for stable operation of future large neuromorphic networks based on such memristors.

## Additional Information

**How to cite this article**: Prezioso, M. *et al.* Self-Adaptive Spike-Time-Dependent Plasticity of Metal-Oxide Memristors. *Sci. Rep.* **6**, 21331; doi: 10.1038/srep21331 (2016).

## Supplementary Material

Supplementary Information
