# Supplementary material for: Self-Adaptive Spike-Time-Dependent Plasticity of Metal-Oxide Memristors
Source: Sci Rep. 2016 Feb 19;6:21331. doi: 10.1038/srep21331 (PMC4759564; doi:10.1038/srep21331)
Supplement: Supplementary Information [file srep21331-s1.pdf]

Supplementary Information

Self-Adaptive Spike-Time-Dependent Plasticity of Metal-Oxide Memristors

M. Prezioso[1], F. Merrikh-Bayat[1], B. Hoskins[1], K. Likharev[2], and D. Strukov[1]

Methods

The device fabrication steps were similar to those described in Ref. 1. Specifically, first, crossbar lines, 200 nm wide and separated by 400 nm gaps, were formed on 4" silicon wafers covered by 200 nm of thermal SiO_2. After standard cleaning and rinse, fabrication started with an e-beam evaporation of Ta (5 nm)/Pt (60 nm) bilayer over a patterned photoresist to form the bottom wires. After liftoff, the wafer was descum by active oxygen dry etching at 200°C for 10 minutes. Then, a blanket film consisting of a 5-nm sputtered Al_2O_3 barrier and a 30-nm TiO_2 switching layer was deposited. This film was then removed by etching in an ICP chamber using CHF_3 plasma, while preserving it in the future crossbar area by pre-deposited negative photoresist. After stripping the photoresist in the 1165 solvent for 3 hours at 80°C, the wafer was cleaned using a mild descum procedure performed in a RIE chamber for 15 seconds with 10 mTorr oxygen plasma at 300 W. Next, the top electrode, consisting of 15 nm Ti and 60 nm Pt layers, was deposited by e-beam evaporation; then top wires were patterned by liftoff process. Finally, the wire bonding pads were formed by e-beam deposition of Cr (10 nm) /Ni (30 nm) /Au (500nm). All lithographic steps were performed using a DUV stepper using a 248 nm laser. After fabrication and dicing, the dies were annealed in a reducing atmosphere (10% H_2, 90% N_2) for 15 minutes at 300°C and wire-bonded to a DIP40 package. The final crossbar layout is shown in Fig.1a.

All electrical characterizations were performed using the Agilent B1500A parameter analyzer with Agilent B1530 arbitrary waveform generator modules. In addition, the Agilent B5250A switching matrix was employed for selecting one device from the crossbar and carrying out the experiment. Before performing the STDP experiments with a memristor, it was electrically

---

[1] Department of Electrical and Computer Engineering, University of California at Santa Barbara, Santa Barbara, CA 93106. [2] Department of Physics and Astronomy, Stony Brook University, Stony Brook, NY 11794.

formed by applying 210 μA, reaching 2.6 V in a current-controlled sweep. After that, the device switching thresholds were measured by applying a double *I-V* ramp (Fig. 1b); they have turned out to be close to 0.7 V for the set operation (i.e. switching from low to high conductive state), and close to -0.8 V for the reset operation.

STDP Modeling

A detailed inspection of experimental data at small positive time delays on Figure 3a explains our particular choice for Λ_t. For example, we have also tried more common expression used in neuroscience

$$\Lambda_T(\Delta t) = \begin{cases} a^{+\prime} e^{-\Delta t/\tau^{+\prime}}, & \Delta t > 0 \\ a^{-\prime} e^{\Delta t/\tau^{-\prime}}, & \Delta t < 0 \end{cases} \quad \text{(S1)}$$

but as Fig. S1 shows such fitting is much less accurate at describing saturation in ΔG at small positive Δt. Such saturation is observed for different values of G_0 and is likely attributed to the specific memristor switching dynamics.

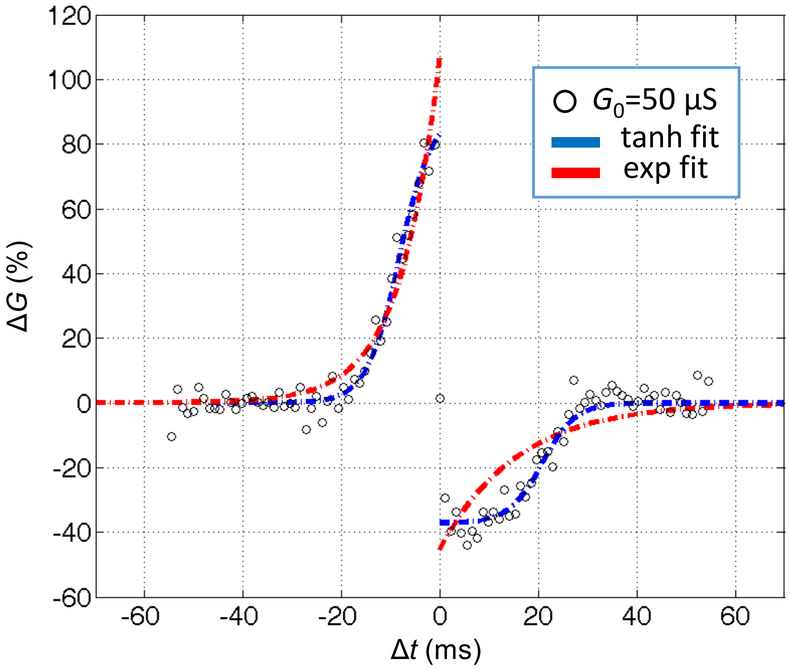

Figure S1. Comparison between the original fitting function that is discussed in the main text (R^2 = 0.9471) and the one described by the same equations but with Λ_t modeled with Eq. S1 (R^2= 0.8756) for the particular case G_0 = 50 μS. Fitting parameters for Eq. S1 are $a^{+\prime}$ = -45.36, $\tau^{+\prime}$ = 0.064, $a^{-\prime}$ = 107.84, $\tau^{-\prime}$ = 0.127.

Self-Tuning Dynamics

To get an additional insight on the convergence dynamics in the considered self-adaption experiment we have studied the influence of the model parameters on the final distribution of memristor conductances. The results of one interesting finding are shown in Figure S2. As this figure shows the smearing and additional hump in the final conductance distributions on Fig. 4d are due to misbalance between a^- and a^+ fitting parameters. Because a^- and a^+ represent, respectively, the potentiation and the depression rate of the synapses for the given STDP window, by increasing a^-/ a^+ ratio more and more synapses evolve, on average, to the higher conductance state. However, when a^- is close a^+ = 13.02, the additional hump disappears and the final conductances are confined within very small range.

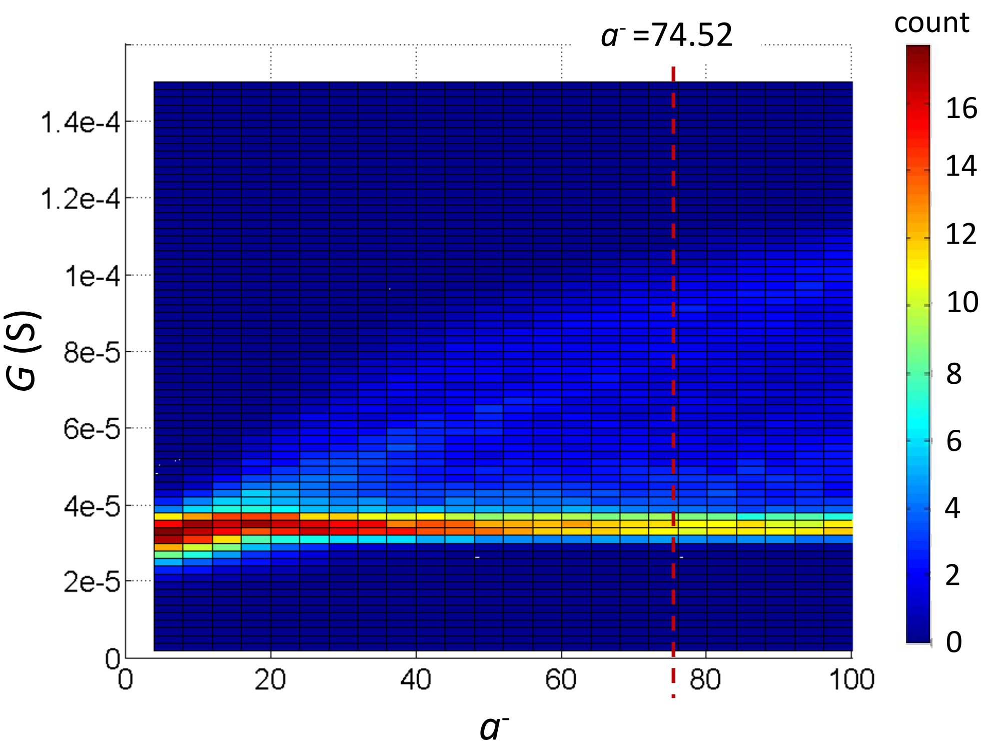

Figure S2. Map of the final distribution of memristor conductances, obtained in the spiking network simulation, using the STDP model described by Eq. (1) – (3), for various choices of the fitting constant a^-. In particular, the plot shown in the middle panel of Fig. 4d is a vertical slice of this map for the particular value a^- = 74.52, shown with the vertical dashed line. The values of other fitting parameters and model assumptions are the same as shown in the inset of Fig. 3b and Fig. 4 of the main text, respectively.

The convergence of the synapse conductance ensemble to an average intermediate state deemed to be important for a practical spiking neural network implementations. For example, had the network converged to high ON or low OFF conductance state distributions under random poissonian stimulation, the neurons would continuously fire without any selectivity or no fire at all.

1 Prezioso, M. *et al.* Training and operation of an integrated neuromorphic network based on metal-oxide memristors. *Nature* **521**, 61-64 (2015).
